# Accuracy of Imputation of Microsatellite Markers from a 50K SNP Chip in Spanish Assaf Sheep

**DOI:** 10.3390/ani11010086

**Published:** 2021-01-05

**Authors:** Héctor Marina, Aroa Suarez-Vega, Rocío Pelayo, Beatriz Gutiérrez-Gil, Antonio Reverter, Cristina Esteban-Blanco, Juan José Arranz

**Affiliations:** 1 Department of Animal Production, Faculty of Veterinary Medicine, University of León, Campus de Vegazana s/n, 24071 León, Spain; hmarg@unileon.es (H.M.); asuav@unileon.es (A.S.-V.); rpelg@unileon.es (R.P.); bgutg@unileon.es (B.G.-G.); cestb@unileon.es (C.E.-B.); 2 CSIRO Agriculture and Food, Queensland Bioscience Precinct, 306 Carmody Rd., St Lucia, Brisbane, QLD 4067, Australia; toni.reverter-gomez@csiro.au

**Keywords:** pedigree verification, sheep, microsatellites, SNPs, marker imputation

## Abstract

**Simple Summary:**

Parentage misassignments directly affect genetic gain in traditional breeding programs. The use of genetic markers facilitates parentage verification. In sheep, microsatellite markers and single nucleotide polymorphism (SNP) markers have been proposed by the International Society of Animal Sciences (ISAG) for parentage testing. Since the implementation of genomic selection, the microsatellite information used for parental testing in previous generations is gradually being replaced by SNPs. However, parentage verifications should all be performed using the same technology. A strategy for transitioning from microsatellites to SNP markers, while avoiding extra genotyping costs, is the imputation of microsatellite alleles from SNP haplotypes. This study aims to identify the optimum approach, using a minimum number of SNPs to accurately impute microsatellite markers and developing a low-density SNP chip for parentage verification in the Assaf sheep breed. The imputation approach described here reached high accuracies using a low number of SNP markers, which supports the development of a low-density SNP chip that could avoid the problems of genotyping with both technologies, being a cost-effective method for parentage testing. This study will help sheep breeders to perform parentage verification when different genotyping platforms have been used across generations.

**Abstract:**

Transitioning from traditional to new genotyping technologies requires the development of bridging methodologies to avoid extra genotyping costs. This study aims to identify the optimum number of single nucleotide polymorphisms (SNPs) necessary to accurately impute microsatellite markers to develop a low-density SNP chip for parentage verification in the Assaf sheep breed. The accuracy of microsatellite marker imputation was assessed with three metrics: genotype concordance (C), genotype dosage (length r^2^), and allelic dosage (allelic r^2^), for all imputation scenarios tested (0.5–10 Mb microsatellite flanking SNP windows). The imputation accuracy for the three metrics analyzed for all haplotype lengths tested was higher than 0.90 (C), 0.80 (length r^2^), and 0.75 (allelic r^2^), indicating strong genotype concordance. The window with 2 Mb length provides the best accuracy for the imputation procedure and the design of an affordable low-density SNP chip for parentage testing. We additionally evaluated imputation performance under two null models, naive (imputing the most common allele) and random (imputing by randomly selecting the allele), which in comparison showed weak genotype concordances (0.41 and 0.15, respectively). Therefore, we describe a precise methodology in the present article to impute multiallelic microsatellite genotypes from a low-density SNP chip in sheep and solve the problem of parentage verification when different genotyping platforms have been used across generations.

## 1. Introduction

Parentage misassignments directly affect genetic gain when pedigree information is used in breeding programs by biasing heritability estimates, genetic parameters, breeding values, and the identification of superior animals for selection [1,2,3]. Therefore, accurate pedigree records are essential for a successful genetic improvement in livestock.

The use of molecular markers, specifically genetic markers, facilitates parentage verification and individual identification by indicating the putative relatedness between individuals through different approaches (simple exclusion, genotype reconstruction, or categorical and fractional allocation) [4,5]. In this sense, microsatellite variants have become one of the principal molecular markers used in livestock in recent decades for parentage testing. Microsatellites, also known as short tandem repeats (STRs) or simple sequence repeats (SSRs), consist of motifs of 1–6 base pairs (bp) repeated in tandem. These variants represent the choice markers for parentage testing in livestock due to their high polymorphic information content with codominant inherited alleles and easy but not fully automated allele scoring [6].

At present, microsatellite information for parentage verification tests is being gradually replaced by single nucleotide polymorphisms (SNPs). Although SNPs are less informative due to their biallelic nature, which determines the range of markers required for parentage testing (200–700 SNPs compared to 14–20 microsatellites) [7], there is increasing interest in using SNP panels in livestock. The advantages of SNP panels include the more straightforward automation of technology, the lack of a need for interlaboratory calibration, lower error rates, the uniform distribution of SNP markers across the genome, and recently reduced costs in genotyping technology [8,9,10]. Moreover, SNP panels are increasingly used in livestock due to the implementation of genomic selection in breeding schemes [11,12,13].

In the case of sheep, there are two strategies for parentage testing proposed by the International Society of Animal Sciences (ISAG): a panel of 19 microsatellites [14] and a panel of 163 SNPs with verified qualities to use in diverse sheep breeds [7]. Notably, in the Spanish Assaf sheep, most of the animals in the selection scheme are genotyped with microsatellite markers. Therefore, the need for a consistent and reliable pedigree database across generations has made the use of microsatellite information an essential issue. However, since the implementation of genomic selection, with the first genomic evaluation results obtained in 2020, there has been an annual increase in the number of animals genotyped with a 50K SNP panel. Some of these animals are genotyped with both platforms: SNPs and microsatellites. Because parentage verification should be performed using the same technology applied in previous generations, this situation has resulted in additional costs for farmers and breeders associations. One possible strategy in the transition from microsatellites to SNP panels to avoid extra costs in genotyping is the imputation of microsatellite alleles from SNP haplotypes [15]. Therefore, in this study, we aimed to identify and evaluate a reliable approach to accurately impute microsatellite markers from an SNP chip panel to perform parental verifications in sheep. Moreover, we evaluated the optimum number of SNPs necessary to accurately impute microsatellite markers to develop a low-density SNP panel for parentage verification.

## 2. Materials and Methods

### 2.1. Animal Genotypes and Quality Control

The genetic profiles of 4423 animals from 94 different flocks included in the breeding program of Spanish Assaf dairy sheep were obtained from the Association of Spanish Assaf Sheep Breeders (ASSAFE, Zamora). This dataset is composed of animals born between 1997 and 2019, of which 349 were artificial insemination rams, 2071 were natural mating rams, and 2003 were ewes. These animals were genotyped for the 19 microsatellites recommended by ISAG for paternity control [14]. A SNP panel with 49,897 markers (50K SNP chip) was used in the genomic selection program implemented in the Spanish Assaf sheep breed. As the data were obtained from the Spanish Assaf breeders association (ASSAFE) database, no direct experimentation on animals was performed in this work. According to the Research Ethics Committee of the University of León, formal ethical approval was not necessary for this case.

Prior to the imputation process, quality control was applied to both sets of markers. We filtered out microsatellites with call rates below 80% and expected heterozygosity below a Hardy-Weinberg equilibrium value of 0.095. This value corresponds to a minor allele frequency (MAF) of 5% for biallelic markers. After quality control, microsatellite alleles were recoded to fit the variant call format (VCF), following the VCFtools software specifications [16]. The most common allele for each microsatellite was considered the reference allele in the population and recoded as “0”. For the rest of the alleles, a consecutive number was assigned (1, 2, 3, …, *n*) based on the microsatellite allele length. SNP-chip quality control was performed using PLINK [17], and SNPs with call rates under 95% were excluded from the dataset. To maintain haplotype diversity in the population, MAF filtering was not included in the SNP quality control.

### 2.2. Imputation Procedure

The positions of the microsatellite markers in the ovine genome (Oar_v3.1) were obtained from the sheep database from Ensembl v.95 (https://www.ensembl.org/Ovis_aries/Info/Index), and they were verified through the alignment of the primer sequences to the sheep reference genome using BLAST [18]. The genotypes were phased and imputed using the phasing method implemented in BEAGLE 5.1 software [19] (50 rounds of burn-in and 100 iterations) and the genotype imputation method [20] of the same program. To establish the minimum number of flanking SNPs per microsatellite and the optimal window length to achieve accurate imputation, several SNP window distances on each side of the marker were considered (0.5, 1, 2, 3, 4, 5, 6, 7, 8, 9, 10, 15, 20, 25, 30, 35, 40, 45, 50 Mega-bases (Mb)). In the genotype imputation process, pedigree information and the effective population size (Ne) were also considered.

Given that the animals were genotyped for both microsatellite and SNP markers, the imputation performance was estimated by a 10-fold cross-validation approach. For this purpose, we divided the total population into two groups: the training group, which comprised 90% of the total population, and the validation group, which comprised the remaining 10%. The microsatellite information was masked in the validation population, and the genotypes for these markers were imputed by Beagle software using all information (microsatellite and SNP genotypes and pedigree relationships from the reference dataset and the genotypes of SNPs in the validation dataset). The process was repeated for ten rounds, using different animals in the validation dataset in each round, following a nonparametric bootstrap of 10% of the total samples using a custom Fortran source code.

### 2.3. Imputation Performance Metrics

To assess the accuracy of the microsatellite imputation, we used the metrics of genotype concordance, genotype dosage, and allelic dosage, which were previously defined by Saini et al. [21]. The genotype concordance ($c_{i}$) was defined as 0 if neither of the imputed alleles matched a true allele, 0.5 if one of them matched, and 1 if both alleles matched the true alleles. Thus, the genotype concordance for a microsatellite (C) was calculated as the average over all the samples of $c_{i}$ for each microsatellite $C=\frac{1}{n}\overset{n}{\underset{i=1}{\sum }}c_{i}$. The microsatellite genotype dosage (length r^2^) was defined as the Pearson correlation between the sum of the two alleles at a specific locus ($d_{i}=x_{i1}+x_{i2}$) in imputed genotypes ($X_{d}=\left\{d_{1},d_{2},\ldots ,d_{n}\right\}$) and the true ($Y_{d}=\left\{d_{1},d_{2},\ldots ,d_{n}\right\}$) genotypes, being the genotype dosage computed as the Pearson correlation of the $X_{d}$ and $Y_{d}$ vectors. The microsatellite allelic dosage (allelic r^2^) was calculated as the Pearson correlation for each microsatellite allele length a, which is defined in the population as $X_{a}=\left\{a_{1},a_{2},\ldots ,a_{n}\right\}$, being *n* the number of samples, where $a_{i}=\overset{2}{\underset{j=1}{\sum }}1_{\left(x_{ij}=a\right)}$, where *j* is the number of alleles per sample. In addition, Pearson’s correlations between the frequencies of the reference microsatellite alleles and the imputed microsatellite alleles were calculated. Furthermore, for each microsatellite, the imputation performance was evaluated by computing the expected value for each metric under two null models: (1) a naive model in which the imputed genotype was selected as the most common allele per microsatellite and (2) a random model in which the imputed genotype was randomly selected from the available genotypes at each marker, depending indirectly on the allelic frequencies [21].

### 2.4. Population Structure, Effective Population Size, and Parental Relationships

Finally, we used SNP chip information to evaluate different factors affecting imputation accuracies, such as population structure, effective population size, and parental–progeny pedigree conflicts. To assess the population structure, we estimated the genomic relationship matrix (GRM) following VanRaden et al. [22], and the genomic relationships between the individuals were plotted following the Pedigromics pipeline [23], calculating centrality metrics such as betweenness and closeness coefficients. In addition, the GRM was also represented, considering the Euclidian distance, and clustered through McQuitty’s criteria using the PermutMatrix software [24]. The effective population size and the parental-progeny conflicts in the pedigree were computed using the BLUPF90 family of programs [25]. Finally, we contrasted the real microsatellite information of the parents, included in the pedigree, with the imputed microsatellite information of the offspring, thus simulating a unique genotyping platform (low-density SNP chip) used in the next generation of individuals. This analysis allowed us to confirm that the procedure proposed here can be applied to impute microsatellite alleles and confirm the parental–progeny relationships.

## 3. Results

### 3.1. Genotype Quality Control

All microsatellite markers passed the quality control settings fixed in the analysis. Regarding SNP markers, a total of 3537 markers showed a call rate lower than 95% and were filtered out. Therefore, a total of 19 microsatellites and 42,665 SNPs were considered for the imputation procedure. The microsatellites were located along the sheep autosomes and on average each microsatellite included in this study had 12.73 alleles, ranging from 81 to 297 bp. The information for the microsatellites considered in this work is summarized in Table 1, and their allele frequencies are presented in Table S1.

### 3.2. Imputation Results

**Figure 1:**
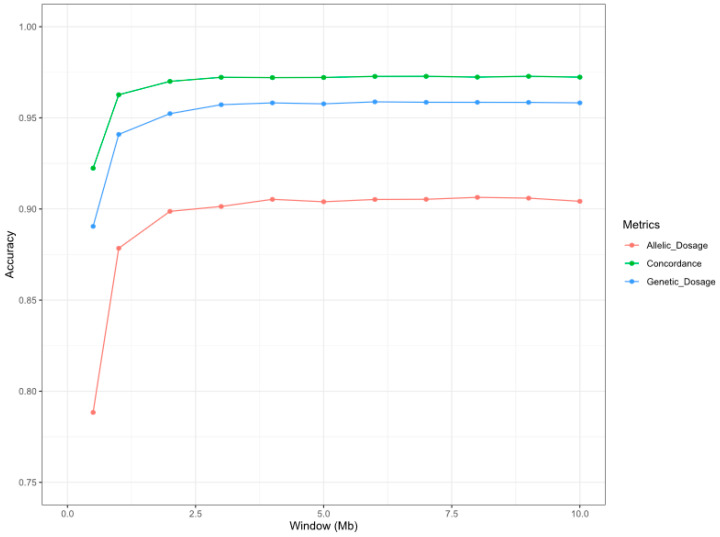
Graphical representation of the accuracy of the microsatellite imputation considering different window lengths in the imputation process. The *x*-axis represents the window sizes (in bp) considered in the imputation process. The *y*-axis represents the average of the imputation accuracy parameters (concordance (green), genotype dosage (blue), and allelic dosage (orange)) of the 19 microsatellites included in this study.

To impute the whole population considered in this study, we performed a 10-fold cross-validation approach, as explained in the Materials and Methods section. Therefore, ten imputation procedures were necessary to estimate the microsatellite information in the whole population. Moreover, we assessed the accuracy (concordance, genotype dosage, and allelic dosage) of the imputed microsatellite markers in the proposed imputation scenarios (window lengths from 0.5 Mb to 50 Mb) to determine the best haplotype length for the imputation procedure (Figure 1).

There was a noticeable increase in the imputation accuracy of the microsatellite markers for the average accuracy metrics when 0.5 Mb (SNPs/window = 19.11, C = 0.922; length r^2^ = 0.890, allelic r^2^ = 0.788), 1 Mb (SNPs/window = 38.05, C = 0.962; length r^2^ = 0.941, allelic r^2^ = 0.878) and 2 Mb (SNPs/window = 74.05, C = 0.970; length r^2^ = 0.952, allelic r^2^ = 0.899) window lengths were compared (Figure 1; Table S2). Considering a 3-Mb window length, the addition of new information provided by the SNPs localized in the surrounding windows (>100 SNPs) slightly improved the imputation accuracy metrics (C = 0.972; length r^2^ = 0.957, allelic r^2^ = 0.901), whereas a stabilization in the imputation accuracy was observed for wider windows (Figure 1). The number of flanking SNPs used in the imputation process for the tested window distances is summarized in Table S3. Considering that the objective of our work was to assess an SNP window length that provides optimum accuracy for the microsatellite imputation procedure to design an affordable low-density SNP chip to be used for parentage testing by breeders, we considered 2 Mb to be the best haplotype for further analyses. The imputation metrics (concordance, genotype dosage, and allelic dosage) for the 2 Mb scenario are summarized in Table 2. The distribution of the allelic r^2^ values is represented in Figure S1. Using a 2 Mb SNP haplotype, the Pearson correlation between the real microsatellite allele frequency in the population and the frequency of the imputed alleles in these markers was 1.00. These frequencies are represented in Figure S2.

To validate our imputation results, we analyzed the imputation performance under two null imputation models: naive imputation, in which imputed genotypes showed an average concordance of 0.41 (ranging from 0.26 to 0.67) with observed genotypes, and random imputation, which had an average concordance of 0.15 (ranging from 0.06 to 0.60). Both validation procedures revealed considerably fewer concordance values using the two null imputation models than the imputation method proposed in this study, which validates our approach.

### 3.3. Population Structure and Effective Population Size

**Figure 2:**
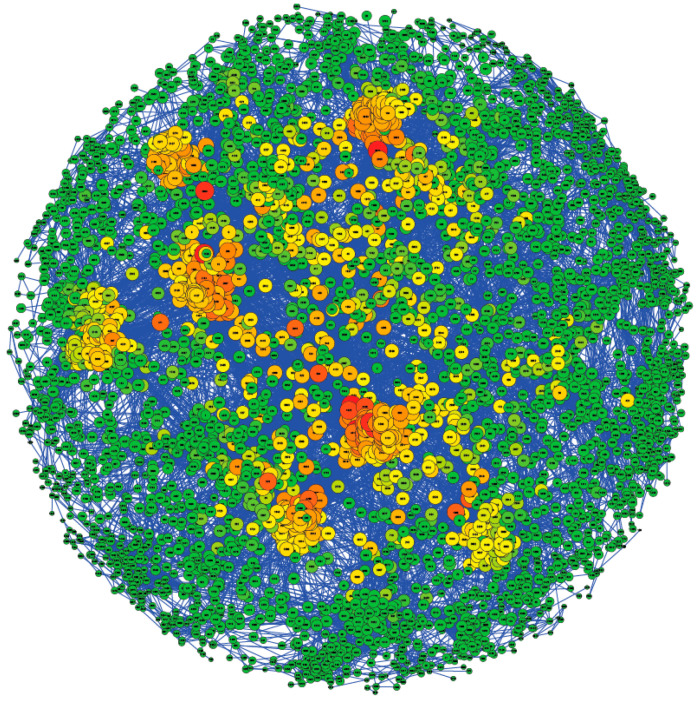
Population structure using the Pedigromics approach. Genomic relationships (>0.2) among the individuals are displayed. Each node represents one animal from the population. The color and the size of the nodes are based on the closeness coefficient, on a green-to-red color scale, with the higher values represented by a large size and red color.

Figure 2 presents the population structure of the 4423 animals included in the study using the GRM created with the 42,665 SNPs remaining after quality control filtering. Individuals are represented as nodes in the network, and two animals are connected by an edge when a pre-defined genomic kinship exists, e.g., parent–offspring. Those animals not related to the main population were filtered in the representation. Genomic relationships higher than 0.2 and 0.5 were represented through edges connecting the animals in Figure 2 and Figure S3, respectively. In addition, the GRM, estimated considering the SNPs remaining after quality control filtering, has also been represented through a heatmap (Figure S4), in which it can be seen that there is not a clear population structure. The Pedigromics approach to the Assaf breed showed low average values of the betweenness centrality coefficient (0.003) and the closeness coefficient (0.237), with both ranging between 0 and 1. Centrality coefficients reflect the influence of each vertex over the graph structure. In this case, closeness centrality is based on the average length of the shortest paths from a given node to other reachable nodes in the network [26], given how genomic information is spread in the population [27]. The betweenness centrality coefficient reflects the amount of control that a node exerts over the interactions with other nodes in the network. Animals with high betweenness centrality in a pedigree graph could have a role in connecting disconnected groups [27]. The low average values of the betweenness centrality and closeness coefficient suggest a low relationship among the samples included in the population studied. However, 21% of the animals had a closeness coefficient higher than the third quartile of the value distribution (0.24), which is represented by a green-to-red color scale in Figure 2. These samples are distributed in eight related family groups (as shown in Figure 2). The low degree of relationship between these groups and the rest of the animals suggests that the population is neither highly related nor structured. Moreover, we estimated the effective population size of the studied Assaf population, which was 214 animals.

### 3.4. Parentage Testing

The pedigree records available for the Assaf sheep population under study integrated 1450 parental–progeny relationships that could be confirmed with the SNP information located in the 2 Mb around each microsatellite to detect parental–progeny conflicts. A total of 24 misassignments were found in the pedigree, representing a total of 1.66% of all the parental relationships analyzed. To ensure that the information from the imputed microsatellites can help verify the relationship between parents and offspring, we contrasted the parental–progeny relationships confirmed through the 19 microsatellites with the imputed microsatellite information, considering the window length scenarios proposed above (1–50 Mb). A total of 86.50% of these relationships were confirmed through analysis of the 19 imputed microsatellite alleles obtained regarding the SNPs located in the 2-Mb surroundings of each microsatellite. Similar values were reached through the 1 Mb (77.50%) and 3 Mb (85.70%) window lengths, with the 2 Mb scenario, described above as the best haplotype for microsatellite imputation, being the best rate in terms of parentage confirmation. The distribution of the compatible microsatellite alleles between parents and offspring is represented in Figure S5, together with the distribution of the average number of compatible microsatellite markers of each individual with the rest of the non-related population. A total of 99.55% of the parental–progeny relationships tested were confirmed considering 17 or more imputed microsatellite markers, as can be observed in Figure S5.

## 4. Discussion

This study presents a precise methodology to impute multiallelic genotypes from biallelic information in sheep. Traditional and new genotyping technologies must be aligned by applying bridge methodologies, which allow breeders to avoid the additional costs of re-genotyping historical data. Our study combines microsatellite and SNP markers in an efficient approach to impute microsatellite markers through SNP haplotypes, achieving high concordance rates. Therefore, the imputation procedure developed represents a useful and inexpensive approach to performing parentage verification when different genotyping platforms have been used across generations. The results from this study will undoubtedly have a great impact on Assaf sheep breeders, allowing them to perform a transition from microsatellite maker kinship verification to the use of SNP panels [28]. In addition to constituting a clear advantage for sheep producers, the imputation methodologies developed can provide advantages in genomic studies by combining both types of data, such as genome-wide association analyses (GWAS). In this approach, microsatellite information could improve the detection of new associations, provide complementary information, and explain part of the missing heritability for the trait under study [21].

In general, as shown in Figure 1, the accuracy of our imputation results for the three metrics analyzed (C, length r^2^, and allelic r^2^) in the different scenarios tested (SNP windows ranged between 0.5 and 10 Mb) was higher than 0.90 (C), 0.80 (length r^2^), and 0.75 (allelic r^2^) for all haplotype lengths. The accuracy results presented in this study were higher than those found in a previous study performed in cattle by Sharma et al. [28], which reached a concordance of 0.40 and a correlation between real and imputed microsatellites of 0.31. In addition, we explored not only the viability of performing microsatellite imputation but also the optimum number of SNPs necessary to perform accurate imputation of microsatellite information. Assessing the accuracy of the imputation of the optimum number of SNPs is crucial to defining an appropriate genotyping strategy to minimize genotyping costs [29]. According to Strucken et al. [7], 700 SNP markers are required to reduce false-positive results in parentage testing, which in our approach correspond to an SNP haplotype length of 1 Mb, covering 38.05 SNPs per microsatellite, with adequate imputation accuracy rates (C = 0.962; length r^2^ = 0.941, allelic r^2^ = 0.878). However, the imputation performance reached high accuracy values at an SNP haplotype length of 2 Mb: 0.97 (C), 0.95 (length r^2^), and 0.90 (allelic r^2^), with all accuracy metrics higher than 0.90. The SNPs located in the 2-Mb window distance used in the imputation procedure have been summarized in Table S4. These results were slightly higher than those obtained in a human genetics study by Saini et al. [21], who achieved a genotype concordance of 0.97, a genotypic dosage of 0.91, and an allelic dosage of 0.86. In our study, accuracy metrics were obtained using a 50K SNP chip in sheep compared to the SNP data from whole-genome sequencing (27,185,239 SNPs) with an SNP window of 100 Kb used by Saini et al. [21]. Moreover, the concordance rates of the null models obtained by Saini et al. (naive (0.72) and random (0.61)) were higher than those obtained in the present study (naive (0.41) and random (0.15)). This highlights the genetic diversity of the microsatellite markers in sheep and the high efficiency of the imputation procedure presented in this work.

The number of haplotypes per microsatellite and the frequency of these haplotypes did not significantly affect the allele dosage, with correlations of 0.33 and 0.18, respectively. Therefore, as the number of alleles and their frequency increases, the concordance tends to rise. However, the correlations between the number of haplotypes of each microsatellite and the concordance rate of the naive model (−0.45) or the concordance rate of the naive model (−0.70) were both negatives, meaning that the naive and random models’ concordance rates decreased as the number of alleles increased because they depended on the number of haplotypes of each microsatellite.

The imputation accuracies obtained may be overestimated due to (i) a highly structured and related population [30] or (ii) a low effective population size [31]. On the one hand, the population included in the present work, represented using the Pedigromics pipeline (Figure 2), achieved low rates of centrality coefficients (betweenness coefficient = 0.003 and closeness coefficient 0.237), which suggests that the population is neither structured nor highly related. In addition, the selection of the reference and test populations during cross-validation using a nonparametric bootstrap approach avoids the overestimation of the imputation metrics by avoiding the selection of immediate relative samples in different groups. On the other hand, the effective population size was 214, higher than in highly selected cattle breeds [28], but in the wide range of effective population sizes described in sheep breeds, from values of 78 in Romney, 100 in the Wiltshire breed, 128 in the Churra breed, to 1317 in Qezel [32,33,34]. Lower values of effective population size can lead to an overestimation of imputation accuracy metrics; however, comparing our concordance rates with the concordance rates obtained in the microsatellite imputation in Hanwoo cattle carried out by Sharma et al. [28], we achieved more than double the concordance (0.90 vs. 0.40). Small population sizes reduce the genetic diversity in the population [35] and would influence the naive and random models’ concordance rates, increasing their accuracy parameters. Nevertheless, the average of the naive and random concordance rates for these two models (0.41 and 0.15, respectively) was far lower than those obtained in a human study by Saini et al. [21], (0.72 and 0.61, respectively). This difference between the imputation accuracy and the accuracy of the null models could be because the effective population size and the genetic diversity of the Assaf population analyzed are large enough to perform an accurate imputation of the microsatellite information. In particular, high genetic diversity in the reference population would help achieve high squared correlations in the imputation process [10,30,36] and reduce the probability of accurate imputations in the naive and random models. Therefore, the large number of samples included in this study, and as a consequence the large number of individuals genotyped in the reference population, could influence the high accuracy rates achieved, because it is necessary to impute the rare haplotypes [31] accurately and this could also reduce the concordance rates obtained in the null models. Therefore, this finding explains the higher concordance rates obtained than those in previous studies on microsatellite imputation from SNP data conducted with lower sample sizes in humans [21] (1916 samples) and Hanwoo cattle [28] (1482 samples).

In summary, the optimum window distance (2 Mb) achieved a high concordance rate (0.97) in the microsatellite imputation procedure and the highest accuracy in parentage testing (>0.99). The parental–offspring relationships confirmed by 17 or more imputed microsatellite markers would ensure a 99.55% success rate, with no risk of parentage misassignments (Figure S5). However, paternity tests with less than 17 imputed microsatellite markers would increase the risk of parentage misassignments and are not recommended in the Spanish Assaf population. These results highlight that 2 Mb is the most appropriate window length for microsatellite imputation and parental verification. Therefore, the development of a low-density SNP panel with the 1407 SNPs (2-Mb SNP window) proposed in this work (Table S4) would also help to reduce the number of kinship errors in the pedigree due to its lower error rates compared with microsatellite markers and the lack of a need for interlaboratory calibration and easier automation [8,9,10].

## 5. Conclusions

This study presents an effective methodology to overcome the problems presented by the transition from multiallelic markers (microsatellites) to biallelic markers (SNPs) for pedigree verification analyses in sheep. The use of a flanking 2-Mb SNP window for each microsatellite has been shown to achieve high accuracy in the imputation procedure while providing a cost-effective, low-density SNP chip for breeders. The microsatellite-imputed information could be used for individual identification and parentage verification in sheep, representing a useful approach in the sheep industry to avoid double genotyping.

## Figures and Tables

**Table 1:** Characteristics of microsatellite markers used in the present study. For each microsatellite marker, the microsatellite ID, genome position (Oar_v3.1), number of alleles per marker, and allele length range expressed in base pairs are shown in the table.

Microsatellite ID	CHR ^1^	Position (bp)	Nº of Alleles	Range (bp)
INRA006	1	109478015	13	104–134
INRA049	1	1952560108	9	134–166
INRA023	1	86986507	14	194–220
FCB20	2	153680836	14	87–115
AE129	5	78045895	6	135–161
SPS113	7	23419543	11	126–152
ILSTS005	7	92854099	12	190–214
ILSTS011	9	25256863	8	268–282
ILSTS008	9	45990219	2	168–170
McM042	9	51865313	8	81–107
CSRD247	14	15564041	19	205–257
INRA063	14	39826970	18	167–207
SPS115	15	23269440	12	237–255
MAF65	15	30901387	9	119–137
MAF214	16	33667802	16	183–269
CP49	17	14434435	25	76–136
HSC	20	25764806	17	263–297
INRA132	20	4668849	17	146–180
INRA172	22	20603037	12	126–172

^1^ CHR = chromosome.

**Table 2:** Imputation performance metrics summary for the 19 microsatellites considered in this study using a 2-Mb window, together with the concordance obtained using the naive and random models.

CHR	Position	Microsatellite	Conc. ^1^	GD ^2^	AD ^3^	Min AD ^3^	Max AD ^3^	Naive Conc.	Random Conc.
1	86986507	INRA023	0.98	0.97	0.97	0.93	0.99	0.28	0.13
1	109478015	INRA006	0.93	0.87	0.84	0.60	1.00	0.48	0.11
1	195256010	INRA049	0.97	0.97	0.88	0.32	0.98	0.44	0.16
2	153680836	FCB20	0.96	0.94	0.89	0.52	1.00	0.26	0.10
5	78045895	AE129	0.96	0.96	0.88	0.13	1.00	0.47	0.20
7	23419543	SPS113	0.95	0.93	0.79	0.16	0.94	0.34	0.16
7	92854099	ILSTS005	0.99	0.97	0.97	0.97	0.97	0.41	0.11
9	25256863	ILSTS011	0.98	0.96	0.92	0.83	0.97	0.50	0.18
9	45990219	ILSTS008	0.97	0.86	0.80	0.37	0.97	0.67	0.61
9	51865313	McM042	0.97	0.97	0.93	0.70	0.98	0.48	0.17
14	15564041	CSRD247	0.99	0.97	0.97	0.94	1.00	0.34	0.07
14	39826970	INRA063	0.97	0.95	0.82	0.47	0.98	0.33	0.09
15	23269440	SPS115	0.96	0.95	0.90	0.67	1.00	0.33	0.14
15	30901387	MAF65	0.98	0.97	0.92	0.75	1.00	0.36	0.18
16	33667802	MAF214	0.98	0.98	0.86	0.32	1.00	0.54	0.09
17	14434435	CP49	0.98	0.97	0.92	0.84	0.98	0.39	0.06
20	4668849	INRA132	0.98	0.97	0.95	0.84	0.97	0.29	0.11
20	25764806	HSC	0.98	0.98	0.95	0.77	0.99	0.54	0.09
22	20603037	INRA172	0.96	0.95	0.91	0.72	1.00	0.35	0.12

CHR = chromosome; ^1^ Conc. = concordance; ^2^ GD = genotype dosage length r^2^; ^3^ AD = allelic dosage allelic r^2^. The definition of each imputation parameter (Conc., GD, AD) is provided in Section 2.

## Data Availability

Restrictions apply to the availability of these data. Data was obtained from the Spanish National Association of Assaf Sheep Breeders (ASSAFE) and are available from the authors with upon request to ASSAFE.

## Supplementary Materials

The following are available online at https://www.mdpi.com/2076-2615/11/1/86/s1, Figure S1: Distribution of the allelic dosage values obtained through the imputation process; Figure S2: The correlation between the minor frequency of the reference short tandem repeat (STR) alleles in the population and the frequency of the imputed STR alleles; Figure S3: Population structure represented through the Pedigromics approach. Each node represents one animal from the population. Genomic relationships (>0.5) among the individuals are displayed. The color and the size of the nodes are based on the closeness coefficient, on a green-to-red color scale, with the higher values represented by a large size and red color; Figure S4: Hierarchically clustered heatmap of the estimated genomic relationship matrix (GRM) considering the single nucleotide polymorphisms (SNPs) created with the 42,665 SNPs remaining after quality control filtering. The genetic relationships are represented through the Euclidian distance on a green-to-red color scale, with the higher values represented in red; Figure S5: Box plot of the distribution of compatible microsatellite markers between parents and offspring considered in this study (left; colored in red) and the distribution of the average number of compatible microsatellite makers of each individual with the rest of the non-related population (right; colored in blue); Table S1: Allele microsatellite frequencies. This table summarizes the length and frequency of each microsatellite allele included in this study in the analyzed population. The allele length is expressed in base pairs (length(bp):frequency); Table S2: Imputation performance summary in the 19 window sizes considered. This table summarizes the average of the imputation metrics (concordance, genotype dosage, and allelic dosage) obtained in the 19 window scenarios considered in this study; Table S3: Number of SNPs located in the different window sizes surrounding the microsatellites. Number of SNPs located in the surroundings of the microsatellite markers for the different window sizes during the imputation process in this study; Table S4: Set of SNPs located in the 2-Mb surrounding of the microsatellites. Worksheet providing the information of the SNPs located at the 2-Mb window distance of the 19 microsatellites included in this study.
