# Supplementary figures and images for: Accuracy of Imputation of Microsatellite Markers from a 50K SNP Chip in Spanish Assaf Sheep
Source: Animals (Basel). 2021 Jan 5;11(1):86. doi: 10.3390/ani11010086 (PMC7824810; doi:10.3390/ani11010086)

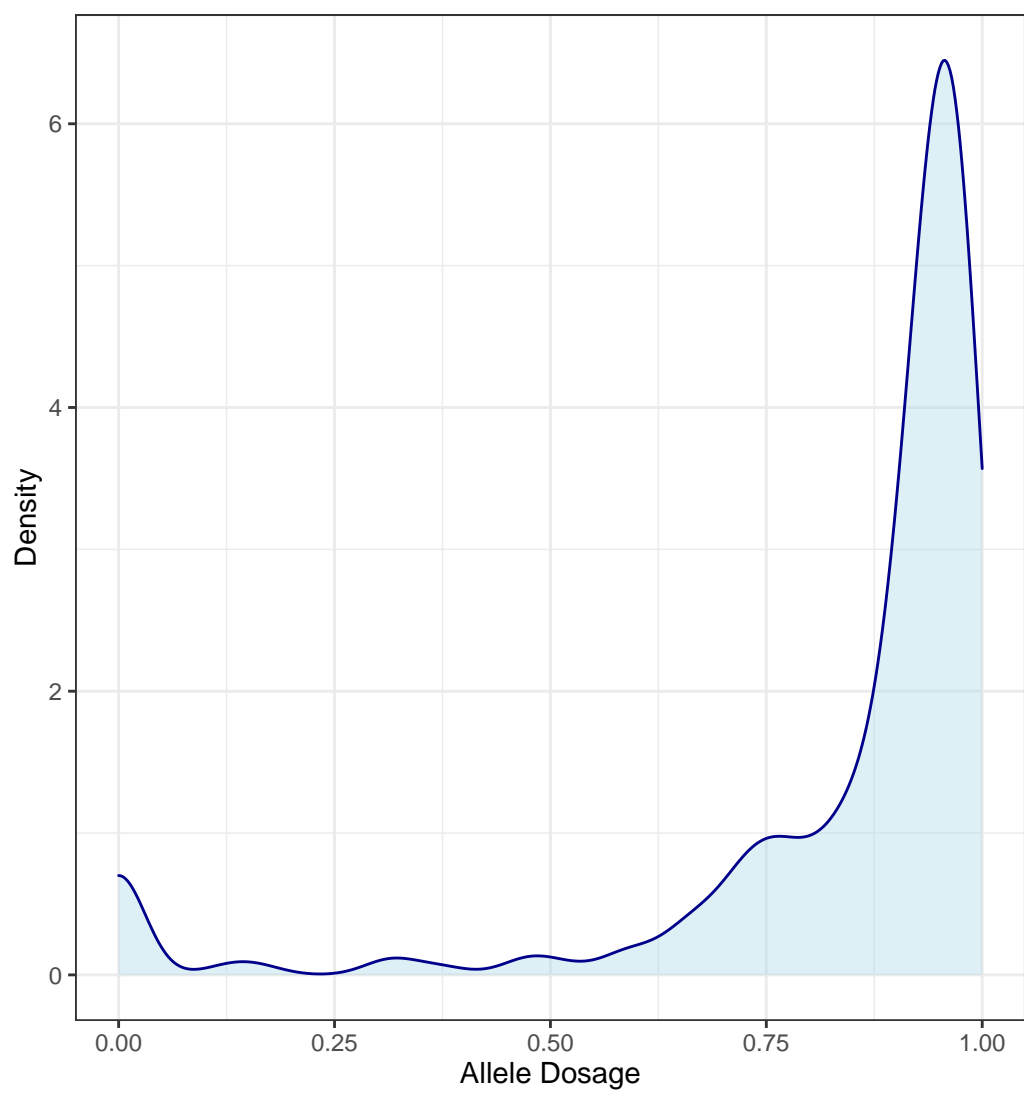

Supplement: Supplementary file 1 [file animals-11-00086-s001.zip › Supplementary Material_animals-103835/Figure S1.pdf]

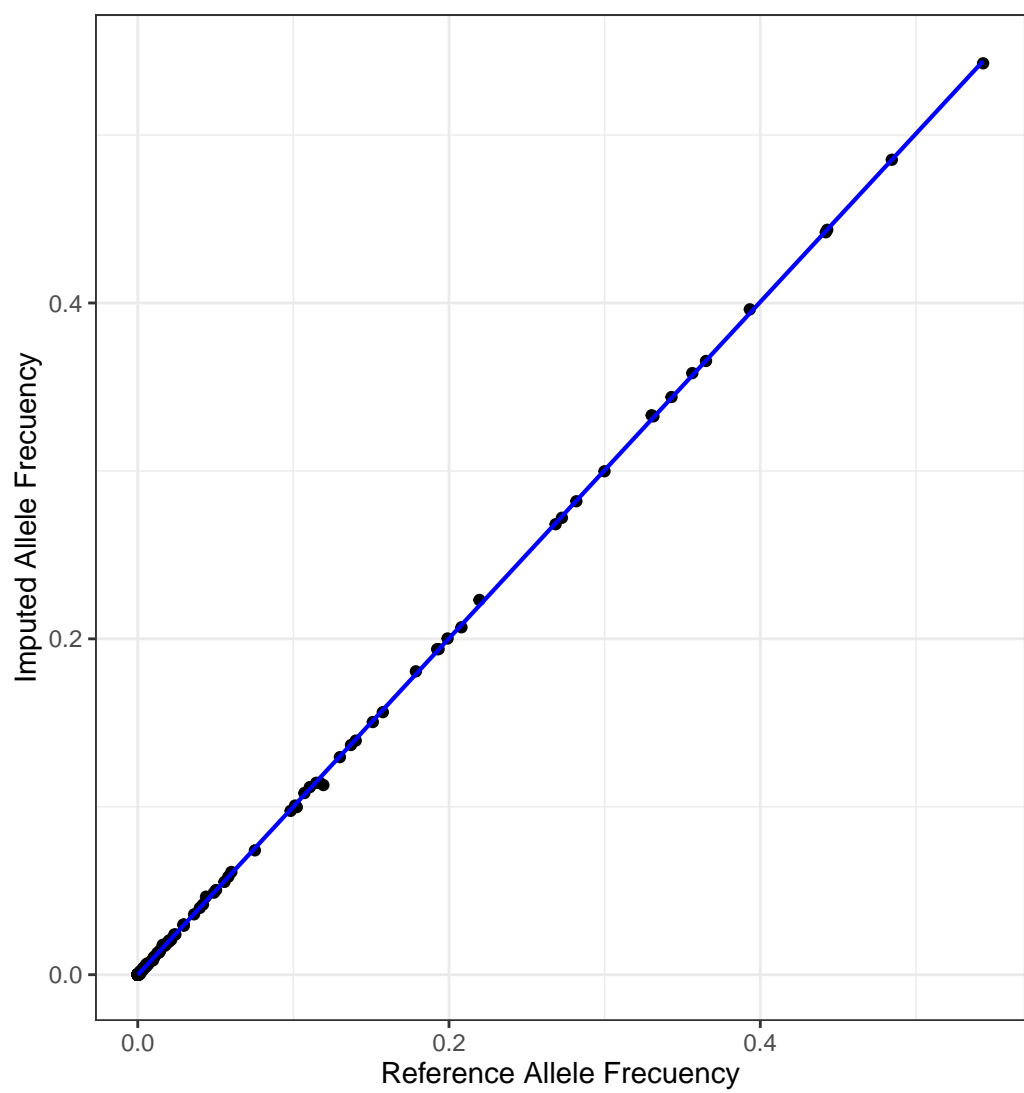

Supplement: Supplementary file 1 [file animals-11-00086-s001.zip › Supplementary Material_animals-103835/Figure S2.pdf]

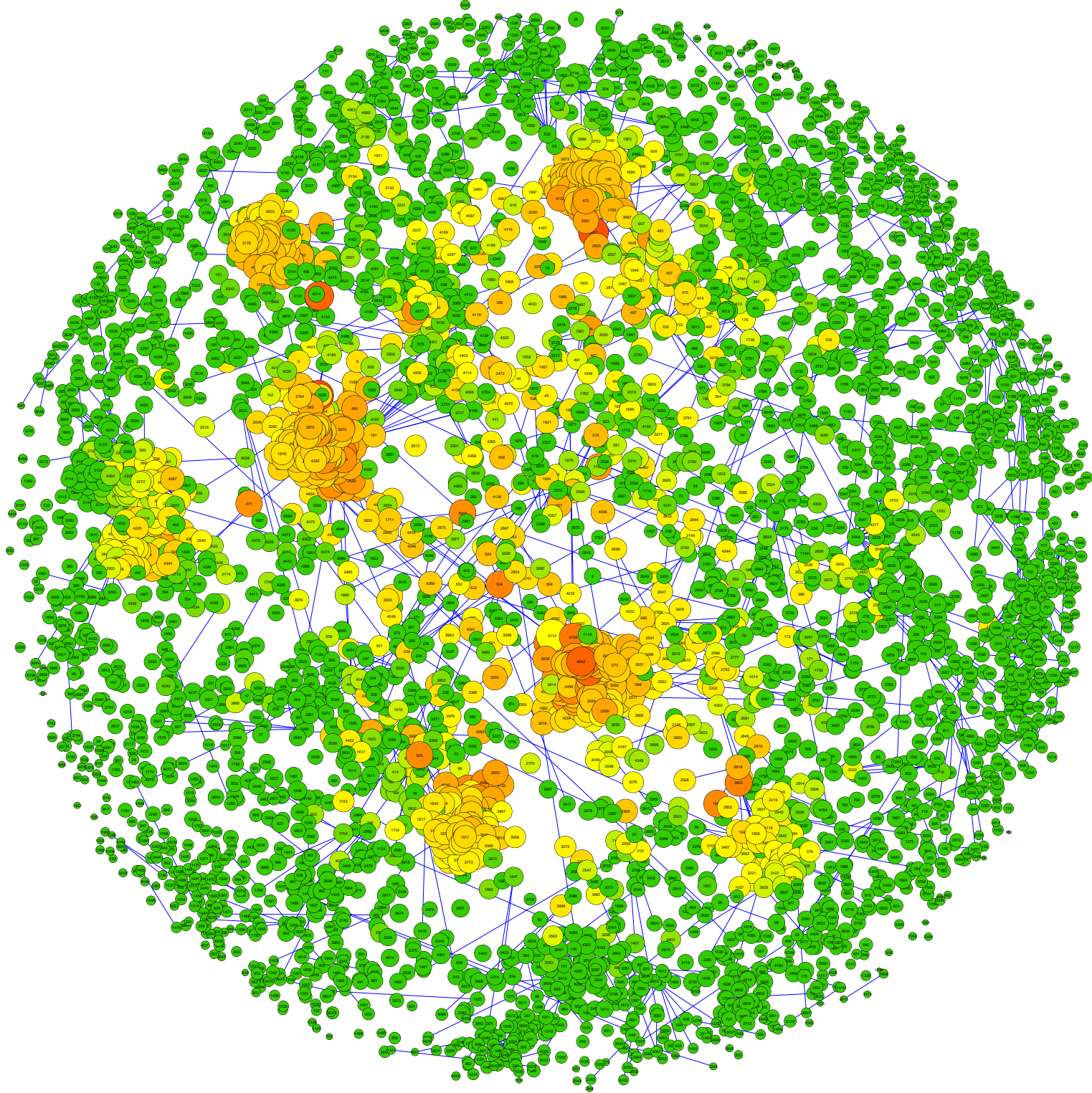

Supplement: Supplementary file 1 [file animals-11-00086-s001.zip › Supplementary Material_animals-103835/Figure S3.pdf]

The colors scale:

Min = -1.39

0.00

Max = 1.39

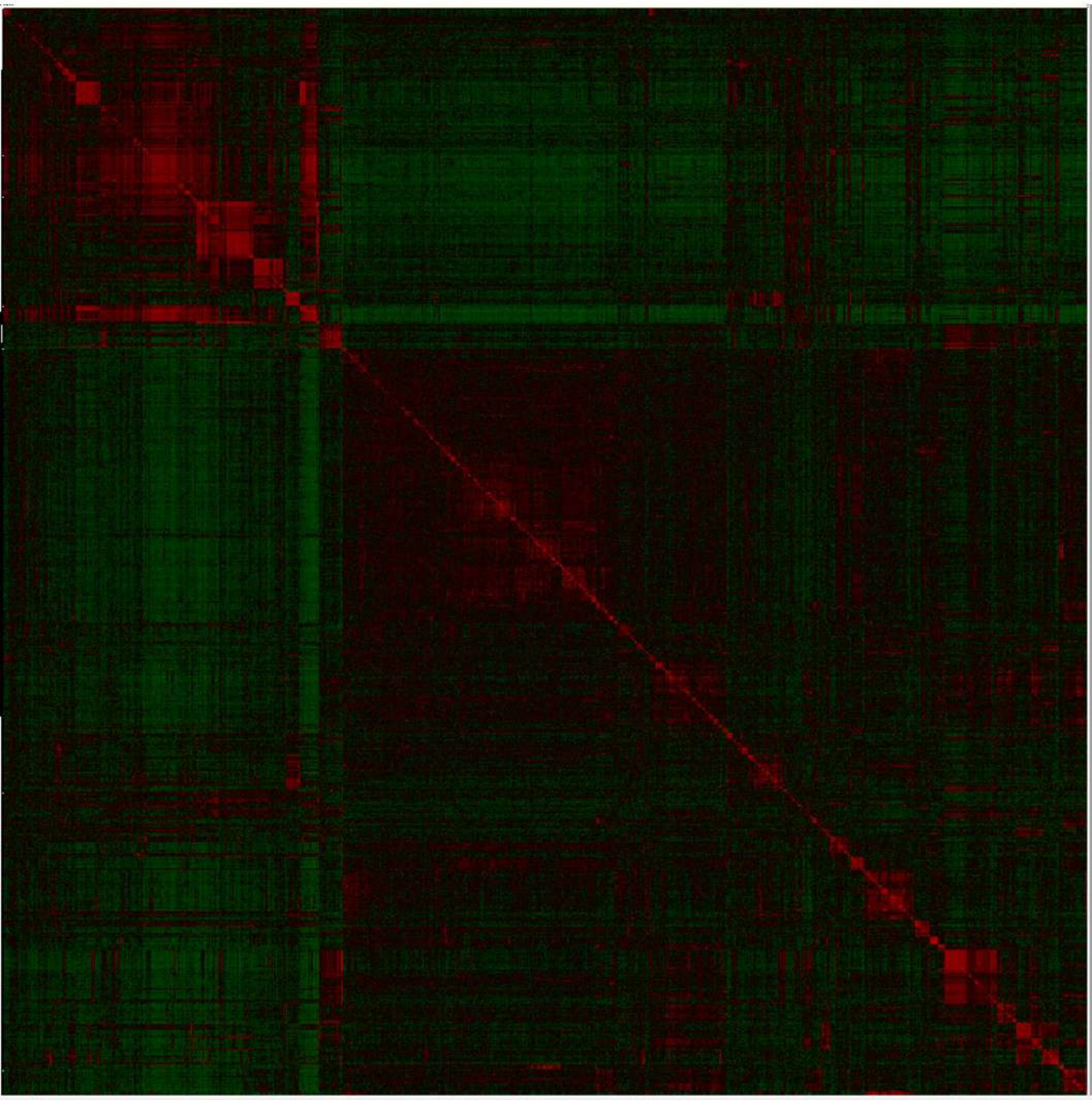

Supplement: Supplementary file 1 [file animals-11-00086-s001.zip › Supplementary Material_animals-103835/Figure S4.pdf]

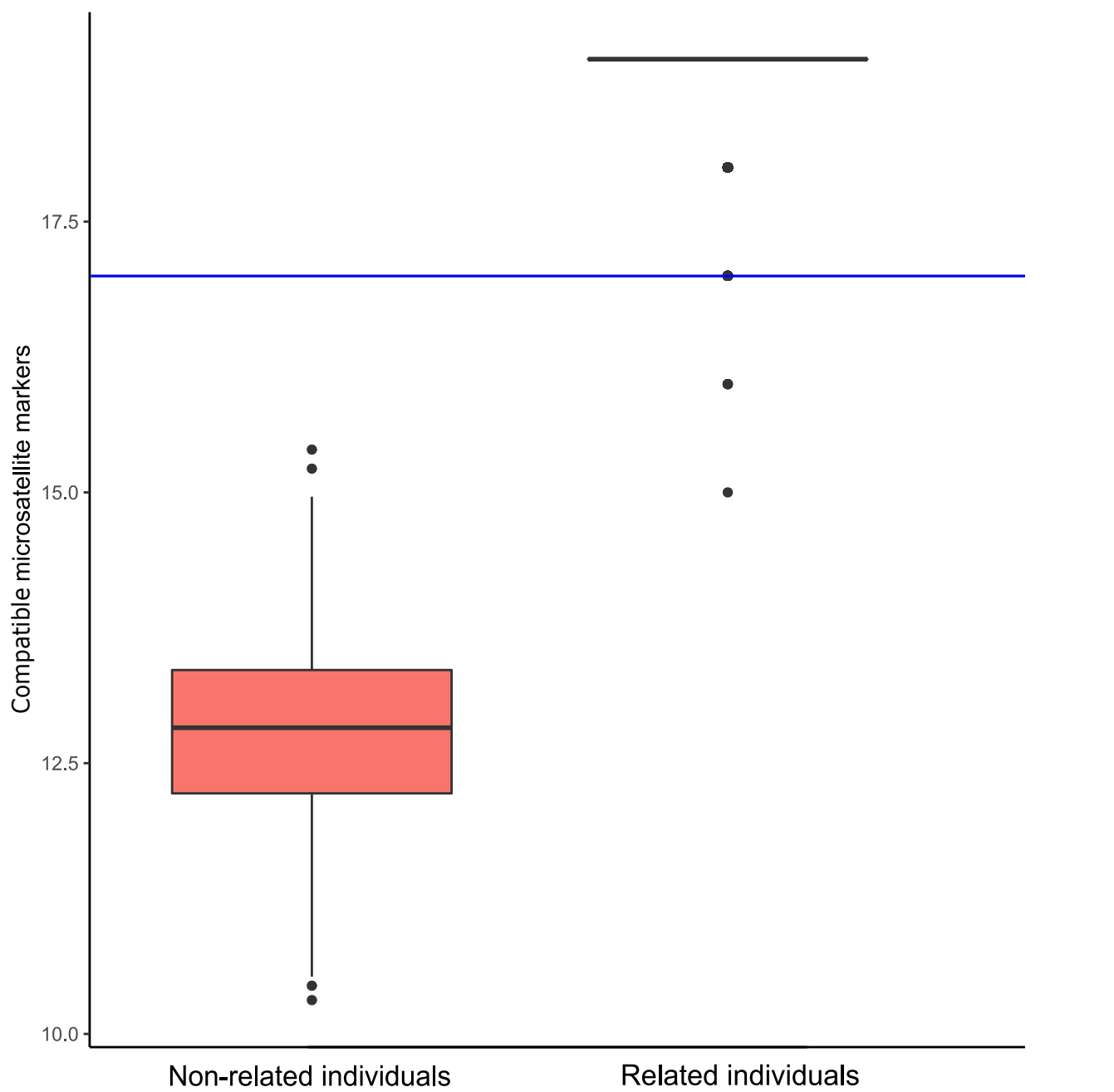

Supplement: Supplementary file 1 [file animals-11-00086-s001.zip › Supplementary Material_animals-103835/Figure S5.pdf]
